# Evaluating official development assistance-funded granting mechanisms for global health and development research that is initiated in high-income countries

**DOI:** 10.1186/s12961-022-00859-6

**Published:** 2022-05-16

**Authors:** Adèle Cassola, Prativa Baral, John-Arne Røttingen, Steven J. Hoffman

**Affiliations:** 1 Global Strategy Lab, York University, 4700 Keele Street, Dahdaleh Building 2120, Toronto, ON M3J 1P3 Canada; 2 Department of International Health, Bloomberg School of Public Health, Johns Hopkins University, Baltimore, 21205 USA; 3 Norwegian Institute of Public Health, 0213 Oslo, Norway; 4 Blavatnik School of Government, Oxford University, Oxford, OX2 6GG UK; 5 Ministry of Foreign Affairs, 0032 Oslo, Norway; 6 Dahdaleh Institute for Global Health Research, School of Global Health and Osgoode Hall Law School, York University, Toronto, M3J 1P3 Canada; 7 Department of Health Research Methods, Evidence & Impact and McMaster Health Forum, McMaster University, Hamilton, L8S 4L8 Canada

**Keywords:** Global health research, Development research, Official development assistance, Research partnerships, Research policy

## Abstract

**Background:**

Several countries allocate official development assistance (ODA) for research on global health and development issues that is initiated in the donor country. The integration of such research within domestic research systems aligns with efforts to coordinate ODA investments with science, technology and innovation policies towards achieving the Sustainable Development Goals (SDGs).

**Methods:**

Through a document synthesis and interviews with research funders in ODA donor and recipient countries, we evaluated the performance of this funding approach across seven donor-country programmes from five donor countries and examined the institutional design elements that increase its chances of advancing development goals and addressing global challenges.

**Results:**

We found that carefully designed programmes provide a promising pathway to producing valuable and contextually relevant knowledge on global health and development issues. To achieve these outcomes and ensure they benefit ODA-receiving countries, programmes should focus on recipient-country priorities and absorptive capacity; translate research on global public goods into context-appropriate technologies; plan and monitor pathways to impact; structure equitable partnerships; strengthen individual and institutional capacity; and emphasize knowledge mobilization.

**Conclusions:**

Global health and development research programmes and partnerships have an important role to play in achieving the SDGs and addressing global challenges. Governments should consider the potential of ODA-funded research programmes to address gaps in their global health and development frameworks. In the absence of concrete evidence of development impact, donor countries should consider making increases in ODA allocations for research additional to more direct investments that have demonstrated effectiveness in ODA-receiving countries.

**Supplementary Information:**

The online version contains supplementary material available at 10.1186/s12961-022-00859-6.

## Background

In recent decades, donor countries’ official development assistance (ODA) policies have become increasingly oriented towards addressing globally interconnected health and development challenges, including through research funding initiatives [1–4]. There has been a documented increase in the share of bilateral ODA devoted to global public goods (GPGs) [1, 5],^1^ and the concept of development as a universal endeavour was reflected in the 2015 Financing for Development Conference, Agenda 2030, and the Sustainable Development Goals (SDGs). The COVID-19 pandemic further highlighted the role of international scientific collaboration and ODA contributions in addressing globally relevant challenges (e.g. [6–8]). At the same time, recent cuts to ODA-funded global health research in the United Kingdom have revealed the challenges associated with sustaining political support for such funding approaches [9, 10].

The formal objective of ODA is to promote economic development and welfare in developing countries [11]. Since 1970, the international community has repeatedly endorsed a target for donor countries of devoting 0.7% of gross national income to ODA [12]. As of 2018, however, only five member countries of the Organisation for Economic Co-operation and Development (OECD) Development Assistance Committee (DAC) met or exceeded this target [13]. Generating and sustaining political support for such spending often depends on demonstrating that ODA creates effective results and also, to some extent, benefits the donor country’s national interests [13–15].

One tool that several DAC member countries have used to achieve development goals both in developing countries and globally involves allocating ODA to fund research that is initiated in the donor country. Such programmes typically aim to enhance donor-country capacity for development research, harness existing capacity to address development challenges and contribute to GPGs, and increase research partnerships with ODA-receiving countries. Although such research programmes currently comprise a small fraction of overall ODA expenditures, knowledge production and capacity-building on these issues can generate strong rates of return and transformative results [16–18]. Moreover, the integration of global health and development research within domestic research systems aligns with efforts in donor countries to coordinate ODA investments with national science, technology and innovation (STI) policies towards achieving the SDGs [19, 20].

The DAC defines research as an ODA-eligible expenditure when it “includes financing by the official sector, whether in the donor country or elsewhere, of research into the problems of developing countries” [21]. However, some concerns remain about the suitability of using ODA for this purpose. In line with the Paris Declaration and Accra Agenda for Action, questions about the impact of ODA-funded research programmes in, and their alignment with the priorities of, ODA-receiving countries are warranted. Decades of studies show that global health and development research initiated in high-income countries can displace local priorities and reinforce unequal power relations; studies also highlight the importance of programme design for ensuring that such programmes benefit lower-income countries (e.g. [22–27]). Similarly, orienting ODA-funded research to areas of universal concern and mutual benefit like GPGs may direct resources away from the localized needs and research agendas of ODA-eligible countries [22, 28–30]. And while research is itself a public good that can theoretically benefit countries of all income levels, they must have in-country capacity to take advantage of new vaccine discoveries or novel technologies [1, 31, 32].

This article evaluates the performance of this research funding mechanism and examines the institutional design considerations that increase its chances of advancing development goals. Although several evaluations and scholarly studies have focused on individual programmes (e.g. [33–35]), to our knowledge, this is the first comprehensive assessment of this funding policy approach across a range of contexts. We conducted a thematic synthesis of programme documents and interviews with government research funders in ODA donor and recipient countries and used a programme evaluation framework to examine the opportunities and challenges associated with this model. We drew our findings together with insights from theories of GPGs provision and critical development studies to formulate recommendations for countries that are interested in adopting or improving this approach.

## Methods

This article draws on qualitative data obtained through a synthesis of 62 documents and interviews with 11 key informants from research funders in ODA donor and recipient countries (eight and three, respectively). This project was reviewed and exempted by York University’s Research Ethics Board. The project methodology description in this section follows the consolidated criteria for reporting qualitative studies [36].

### Programme selection

To maximize the comparability of evaluated programmes, we established three selection criteria. Qualifying programmes were funded primarily through ODA; provided ODA funding to domestic (donor country) researchers; and were delivered through domestically focused donor country government research funders. We identified programmes through extensive internet searching and contacts at research funding bodies and international organizations. We also asked personnel at the programmes we identified to list similar programmes. We considered the list complete once additional inquiries did not yield new suggestions. The programmes or divisions we identified were as follows:

- Science for Global Development (WOTRO) (Netherlands)
- Global Health and Vaccination Research (GLOBVAC) (Norway)
- Norway—Global Partner (NORGLOBAL) (Norway)
- Program for Development Research (Sweden)
- Program for Research on Global Issues for Development (r4d) (Switzerland)
- Global Challenges Research Fund (GCRF) (United Kingdom)
- Newton Fund (United Kingdom)

We did not include programmes delivered through government agencies or institutions that are primarily externally oriented, such as Canada’s International Development Research Centre and France’s Institut de Recherche pour le Développement. Similarly, we did not include programmes that allocate ODA for expenses associated with conducting research in ODA-eligible countries but use other funding sources to support the portion of research conducted outside of the ODA-receiving country (e.g. Japan’s Science and Technology Research Partnership for Sustainable Development). While these and other programmes often have similar aims to those included in our evaluation, narrowing our focus enabled us to more accurately evaluate the role of institutional design among programmes that share basic similarities in funding and administrative structures.

### Analytical framework

The document review and interviews were guided by a programme evaluation framework adapted from public administration theory [37, 38] (Table 1). Certain evaluative criteria were additionally informed by themes on North–South power relations, localized priority-setting and GPGs, as discussed in the introduction of this article.

**Table 1:** Framework for evaluating ODA-funded development research programmes

Domain	Key questions	Institutional design considerations
Effectiveness	• Has this funding mechanism changed the research landscape in donor and recipient countries?	• What are the programmatic strengths, weaknesses and avenues for improvement?
	• How does the effectiveness of this approach compare to more direct ODA allocations for programmes and services in recipient countries?	• What design elements contribute to, or could increase, its effectiveness?
	• Are there any unintended negative consequences associated with this approach?	• Could programme design elements help to avert these unintended negative consequences in the future?
Efficiency	• Is this research funding approach a cost-effective way to make progress on global health and development goals?	• Could the approach be designed to achieve greater value for the money?
	• Does this funding approach increase coordination between the research community and policy/development actors?	• Are there any additional strategies that could promote such coordination?
Equity	• Does the programme design promote equitable research partnerships in ODA-receiving countries, and if so, how?	• How can specific rules around data-sharing, intellectual property rights and authorship be designed to increase equity?
	• Does the programme design promote sustainable capacity-building in ODA-receiving countries, and if so, how?	• What design elements can support continued capacity-building after the initial funded research is completed?
Political feasibility	• What kind of political support or opposition has the approach encountered in donor countries?	• What programmatic elements have made the approach more or less attractive politically in donor countries?
	• How is this research funding mechanism perceived by the development and research communities in ODA-receiving countries?	• What programmatic elements have made the approach more or less attractive in recipient countries?
Management processes	• How are programme priorities set?	• How do different actors and institutions influence the process?
	• What is the operational burden of these research programmes?	• How many full-time staff are required to deliver such programmes?

### Document review

We analysed publicly available documents that provided descriptive and evaluative information about each programme, including programme brochures, annual reports, evaluations, website descriptions, rapid reviews, calls for proposals and research articles. Documents were collected between November 2018 and August 2019 by two researchers (AC/PB). We identified relevant documents through programme contacts and by searching programme websites, academic repositories and the grey literature. Evaluations of sub-programmes or initiatives under the purview of the included programmes were also reviewed. When multiple versions of similar documents were available for the same programme (e.g. annual reports or calls for proposals), we included the most recent version available. A total of 62 documents were identified for our analysis.^2^ The majority of documents were available online, and two additional documents were obtained through programme contacts. Once the documents were collected, two team members (AC/PB) extracted information about programme objectives, operational features and outcomes, and synthesized findings using our evaluative framework.

### Key informant interviews

We conducted interviews with a purposive sample of key informants from ODA donor and recipient countries. In donor countries, we interviewed senior individuals involved in managing each programme; they were identified through existing contacts and programme websites.^3^ We identified key informants at research funding bodies in ODA-receiving countries through the expertise of the Council on Health Research for Development (COHRED).^4^ We interviewed one individual in each of three countries representing different regions and income levels. Interviewees were associated with the Oswaldo Cruz Foundation (FIOCRUZ) in Brazil, the Philippine Council for Health Research and Development, and the Tanzania Commission for Science and Technology.

The interview questions (Additional file 1: Appendices S1 and S2) were informed by our evaluative framework and piloted with an individual in a relevant position at a research funder in an ODA donor and recipient country, respectively. Interviews aimed to understand the funding model’s performance in achieving development goals and the institutional design features that influenced effectiveness, efficiency, equity and political feasibility. Donor country interviews also included questions about programme management. Interviewees were recruited by email and received a description of the project as well as the interview guide in advance. Interviews were conducted by AC (female, PhD) in 2019 over telephone or web conferencing and lasted between 25 minutes and 1 hour. Interviews were audio-recorded with consent. In the “Results” section, interviewee quotes from ODA donor countries are referred to using the codes ODADC 1–8 and those from ODA-receiving countries are referred to using the codes ODARC 1–3.

### Interview data analysis

We conducted a thematic synthesis of the interview data using Yin’s five stages of qualitative analysis (compiling, disassembling, reassembling, interpreting and concluding) [39]. We first imported the interview transcripts into NVivo software, reviewed them and extracted descriptive information. One team member (AC) subsequently coded salient concepts in the interviews, identified patterns of responses and grouped concepts together to elucidate broad themes. Although our evaluative framework (Table 1) structured the coding process, we did not have predefined hypotheses about what the data would show and used an inductive approach to identify emergent themes. During the coding process, each of the theoretical lenses that guided the analysis yielded different insights. The programme evaluation lens foregrounded questions about programme performance using traditional criteria of effectiveness, efficiency, equity, manageability and political feasibility; the GPGs lens foregrounded questions about programmes’ broader potential as a tool to address global challenges; and the critical development lens foregrounded questions about programme partnerships and impact from the perspective of ODA-receiving countries. The major themes and their significance were discussed among the project team at key junctures in the analytical process, and these conversations generated new insights that informed additional data review and verification. We organized our narrative synthesis of results around key themes regarding programme performance and institutional design and then integrated broader insights from the critical development and GPGs literature to conclude our analysis with policy recommendations. To ensure the accuracy and validity of our findings, interviewees had the opportunity to read and comment on the manuscript [36].

## Results

### Descriptive summary of the funding mechanisms

The document review and interviews confirmed that the included programmes shared several similar components (Table 2). Each programme had domestic goals (e.g. increasing national development research capacity); aims oriented towards ODA-receiving countries (e.g. increasing research collaboration); and areas of mutual benefit (e.g. enhancing knowledge to address SDGs). In line with the ODA definition, all programmes focused on research relevant to the development of ODA-eligible countries. The programmes also relied on a similar cast of actors, including the research funders operating programmes, ministries that provided funding, domestic higher education institutions, and institutions in partner countries.

**Table 2:** Key features of ODA-funded granting mechanisms

Objectives include…	Increasing domestic interest and leadership in global health and development research Enhancing knowledge about countries with increasing global influence Improving the basis for bilateral research cooperation Increasing research collaboration with, and capacity of, partners in LMICs^a^ Supporting work of development agencies and policy-makers Harnessing research expertise to contribute to GPGs and the SDGs
Key institutions include…	Government research funders National development agencies Foreign affairs ministries Domestic higher education/research institutions LMIC higher education/research institutions (when programmes involve partnerships) LMIC governments (when programmes involve co-funding or joint priority-setting) OECD Development Assistance Committee (DAC)
Broad substantive focus	Economic development and welfare of developing countries
Broad geographical focus	OECD DAC list of ODA-eligible recipient countries

^a^Low- and middle-income countries

Aside from broad institutional similarities, programmes differed in size and structure (Table 3). Average yearly budgets ranged from US$ 7.9 million to US$ 405 million; when expressed as a percentage of each country’s 2018 ODA expenditure, the figures ranged from 0.19 to 2.06%. Interviews revealed that personnel resources also varied, with full-time equivalent staff ranging from 1.5 to over 50.

**Table 3:** Comparison of ODA-funded programmes for global health and development research initiated in donor countries

Programme/department name	Netherlands	Norway		Sweden	Switzerland	United Kingdom	
	Science for Global Development (WOTRO)	Global Health and Vaccination Research (GLOBVAC)	Norway—Global Partner (NORGLOBAL)	Program for Development Research	Research on Global Issues for Development (r4d)	Global Challenges Research Fund (GCRF)	Newton Fund
Managing research funding body	Netherlands Organisation for Scientific Research	Research Council of Norway	Research Council of Norway	Swedish Research Council	Swiss National Science Foundation	UK Research and Innovation (UKRI) Research Councils, academies, space agency and funding councils	UKRI, United Kingdom academies and Met Office
Programme budget in local currency	~ EUR 70 million 2013–2020 ~ (EUR 10 million annually)^a^	NOK 65 million annually 2018–2020	~ NOK 569 million 2016–2023 (~ NOK 90–100 million annually)^b^	~ SEK 183 million annually	CHF 97.6 million over 10 years (CHF 9.76 million annually)	GBP 1.5 billion over 5 years (GBP 300 million annually)	GBP 735 million 2014–2021 (GBP 105 million annually)
Average yearly budget in USD (2017 rate)	~ 12 million	7.9 million	~ 11–12 million	~ 22.3 million	10 million	405 million	142 million
Percentage of country’s 2018 ODA^c^	~ 0.21%	0.19%	~ 0.29%	~ 0.36%	0.32%	2.06%	0.72%
Period of operation	WOTRO division founded in 1964	Period 1: 2006–2011 Period 2: 2012–2020	Period 1: 2009–2014 Period 2: 2016–2023	Programme established in late 1970s	Programme period 2012–2022	Programme period 2016–2021	Programme period 2014–2021
Eligibility	Some calls require lead applicants from the Netherlands; others invite lead applicants from LMICs	Norwegian institutions must be project owners. Principal investigator can be from outside Norway	Norwegian research institutions must be primary applicants	Grants must be administered by a Swedish institution	Researchers at Swiss or LMIC institutions are eligible but Swiss institution must be lead applicant	Varies by call/delivery partner	Projects require a lead in the United Kingdom and a lead in the partner country
Type of partnership with ODA-receiving country researchers	Required	Encouraged	Required	Varies by call for project grants; required for network grants	Required	Varies by call	Required and developed at government level

Information was obtained in 2019 through programme documents, interviews and correspondence with key informants

^a^Estimate refers to the ODA-funded programmes that WOTRO manages on behalf of the Ministry of Foreign Affairs

^b^Annual estimate based on allocations to date at time of analysis

^c^2018 ODA net disbursements in national currencies were extracted from OECD statistics on total flows by donor

Although they were all managed by domestically oriented government research funders, the programmes varied in their delivery structures. For example, WOTRO is a division of the Netherlands Organisation for Scientific Research (NWO) that oversees several programmes, some of which are ODA-funded and managed on behalf of the Ministry of Foreign Affairs.^5^ While the Swiss National Science Foundation is responsible for the operational delivery of r4d, the programme is a joint partnership with the Swiss Development Corporation. The delivery of the GCRF and Newton Fund is spread across multiple partners. The Newton Fund also takes a different form from the other programmes by focusing on bilateral collaboration with middle-income countries and including partner-country governments in project development, funding and delivery.

Programmes also differed with respect to the rules governing partnerships with researchers from ODA-receiving countries. Four programmes required such partnerships (WOTRO, NORGLOBAL, r4d and the Newton Fund), one encouraged them (GLOBVAC), and in two cases, requirements varied across calls for funding (GCRF; Sweden’s programme for development research). Moreover, programmes took different approaches to whether a donor country institution was required to be the lead applicant for funding. This was the case for at least some calls in each programme, although details varied. For example, the Newton Fund was co-funded by the United Kingdom and partner governments and required a lead in each country, with United Kingdom ODA funding flowing through United Kingdom institutions and spent primarily within the United Kingdom [40]. The r4d programme required Swiss institutions to be lead applicants, but also required projects to be developed and led together with developing-country partners, at least half of project academic personnel to be in developing countries, and at least 40% of project funding to be allocated to these partners (e.g. [41, 42]).

### Synthesis of previous programme evaluations and reports

This section synthesizes the major findings and recommendations of past programme evaluations and reports. Overall, the funding model was found to have positively impacted donor countries’ research landscapes, but direct impacts on ODA-receiving countries were less evident. A second recurring finding involved the challenges of developing partnerships that are equitable and support sustainable capacity-building, and the importance of institutional design for improving performance in this area.

#### Findings on programme effectiveness

Past programme evaluations and reviews reported several positive effects on research landscapes, with most evidence related to donor countries. Impacts included attracting domestic researchers and institutions to global health and development research; promoting the prestige, quality and interdisciplinarity of such research; increasing the volume of studies and publications in this field; enhancing policy-makers’ knowledge of and support for such research; and increasing and strengthening international research partnerships [40, 43–48].

However, direct evidence of contributions to the objective of ODA—improving economic development and welfare of developing countries—was less common. Some examples did emerge: for example, an evaluation of r4d, a programme that requires researchers to set aside 10–15% of their grant for application and dissemination activities, found that “all sampled projects have brought emerging results into policy fora and among stakeholders” [45]. Evaluations of GLOBVAC and the Newton Fund also identified several projects that had produced outcomes with direct relevance to developing countries [40, 43]. Overall, however, direct development-related outcomes were not commonly reported. Past evaluations outlined several challenges associated with identifying and achieving such outcomes, including the long-term nature of many research projects and the lack of sufficient mechanisms for planning and monitoring impact in some programmes. For example, a review of the GCRF noted the challenge of ensuring that “research continues to be ODA-compliant over the lifespan of the project”, especially since the applications of basic scientific research may take time to emerge [44]. Across programmes, reports also identified tensions between encouraging policy-relevant projects with immediate results and the long time frames required to find solutions to complex development challenges [43–45, 47, 49, 50].

One avenue for increasing programmes’ development impact that was identified in several evaluations involved translating research into relevant knowledge for policy-makers and other actors. While such exchanges were described as a key pathway to effectiveness, they were often found to be lacking in practice. Evaluators’ recommendations for improving performance in this area included requiring applicants to integrate a dissemination strategy into their proposals and progress reports, establishing built-in channels of communication between funders and development agencies, and creating opportunities for development agency involvement in translating research to policy [44, 45, 50–52].

#### Findings on equitable partnership development and capacity-building

The design and impact of programme partnerships also emerged as a key theme from past evaluations. Partnerships between ODA donor and recipient country researchers were described as important for various reasons; in addition to equity-based considerations, collaboration was found to improve research relevance, quality, manageability and usability and to contribute to capacity-building in ODA-receiving countries [43, 45, 50, 53]. At the same time, forging equitable partnerships was identified as a challenge across several programmes. Programme-level imbalances such as requiring donor country institutions to be lead applicants and allocating funds through them, as well as project-level cases where ODA-receiving country researchers were only included in data collection and analysis and were not part of the publication team, affected partnership equitability and opportunities for capacity-building [43–45, 50]. For example, an evaluation of r4d identified the flow of funds to partners through Swiss institutions as “inherently asymmetrical” [45]. In the United Kingdom, the equity implications associated with dedicating a substantial share of ODA funds to donor country institutions have also been questioned, as this practice may violate the spirit of the government’s commitment to untied aid [33, 40, 54].^6^

Programme design features that were recommended to advance equitable partnerships and promote capacity-building included lengthening application periods to facilitate the development of new international partnerships; enhancing opportunities for project ownership by ODA-receiving country partners; allowing joint applications between ODA donor and receiving country researchers; providing support for partners to co-develop proposals; dedicating funding for ODA-receiving country partners to develop applications and publish research; increasing opportunities for research partners to work in each other’s contexts; and providing training on equitable partnership guidelines [43–45, 50, 51, 56]. Evaluations also noted the potential for programmes to promote longer-term capacity-building by strengthening institutions in addition to facilitating individual knowledge transfer and by coordinating with other capacity-building initiatives [40, 43].

However, evaluations also identified trade-offs between promoting effective partnerships that produce high-quality research and building capacity in more challenging settings. For example, an r4d evaluation noted that collaborations were more effective when partners had existing working relationships and matched or complementary abilities, and knowledge users described better access to and alignment of research “where developing-country partners had high convening power, strong scholarly reputations, and undertook proactive engagement” [45]. The Newton Fund, which has capacity-building as a main goal, has focused support on higher-capacity partner countries and institutions; the co-funding design has been identified as disadvantaging countries with lower research and innovation capacity [40].

### Analysis of interviews with government research funders

Interviews with research funders in ODA donor and recipient countries shed further light on programme performance and the institutional design elements that affect key outcomes. Table 4 summarizes the five major findings we identified from the interviews. Table 5 categorizes the combined findings from the interviews and document review along our evaluative dimensions.

**Table 4:** Key findings derived from the analysis of interviews

1. Pathways to impact are often indirect, and evidence of long-term outcomes is difficult to obtain
2. Delivering programmes through and with research funding bodies can increase effectiveness and efficiency
3. Partnerships are more effective when equity is built into programme and funding rules
4. Capacity-building is often donor country-focused and happens more indirectly in partner countries
5. Programme priority-setting processes are often top-down and donor-led, but call- and project-level priorities are more flexible and opportunities exist to pursue mutual goals

**Table 5:** Evaluation of ODA-funded development research model based on interview data and document review

	Summary of findings	Potential pitfalls	Promising practices
Effectiveness	• Pathways to impact are often indirect and long-term • Development impacts are difficult to quantify and are not always strategically planned or adequately tracked	• Weaker capacity may prevent ODA-receiving countries from applying new knowledge and technologies • Donor country policy-makers may question the value of research programmes when concrete results are not evident • Funded projects may lack direct relevance to ODA-eligible issues	• Maximizing participation from ODA-receiving countries can enhance development relevance • Collaboration with ODA-receiving country partners can increase capacity-building outcomes • Strategic planning, monitoring and evaluation mechanisms should be built in to maximize development impact and ensure ODA compliance
Efficiency	• Programmes delivered by research funders can produce high-quality projects and increase the prestige of development research • Research can provide an evidence base for the work of development actors	• Redundancies may emerge among funders working on the same topic or in the same partner country • Research may fail to be translated into knowledge that development actors use	• Coordinating with research funding institutions in recipient countries can increase efficiency • Regular channels for exchange should be established among researchers, policy-makers and development actors
Equity	• Programmes have increased partnerships between ODA donor and recipient countries • Capacity-building in ODA-receiving countries can help to address structural inequalities	• Partnerships can be imbalanced from the outset due to funding arrangements and priority-setting processes • A lack of resources may prevent donor country research councils from addressing capacity-building in recipient countries directly • Long-term capacity-building may not be sustainable once project funding expires	• Structures to ensure equity should be built into programmes • Additional provisions on equity should be included in project calls, review processes and partnership agreements • Capacity-building goals should be made explicit and supported by specific programme provisions • Projects’ potential for continued impact should be assessed during the application process
Political feasibility	• Donor country policy-makers need evidence of impact to understand programme value • Recipient countries welcome programmes that include collaboration, co-development and capacity-building	• Politicians may not see the value in supporting research programmes that have longer timelines for results than the shorter-term electoral cycles • Co-funding models may be a challenge in ODA-receiving countries where resources for research are limited or under pressure	• Concrete examples and instances of international uptake can demonstrate value to policy-makers • Donor countries should take careful stock of the financial and political situation in partner countries and, with their input, design programmes accordingly
Management processes	• Broad priorities are often set by donor country ministries, but research funders have flexibility at the call level	• Orienting development research to GPGs and other areas of mutual benefit may displace local development agendas	• Input from ODA-receiving countries should be integrated at as many levels as possible during programme, call and project development

#### 1: Pathways to impact are often indirect, and evidence of long-term outcomes is difficult to obtain

**Fig. 1:**
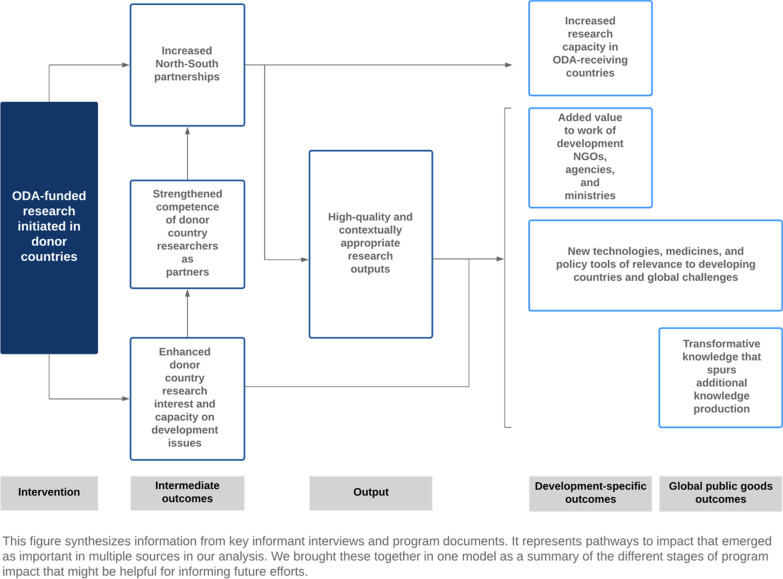
Pathways to impact from ODA-funded research initiated in donor countries to development and GPGs outcomes (derived from interview and document data)

We asked interviewees to assess how ODA-funded research programmes had changed the research landscapes in their countries, to describe the model’s effectiveness in achieving development goals compared with more direct aid investments, and to evaluate the cost-effectiveness of this use of ODA. Interviewees identified numerous pathways to impact (Fig. 1). Consistent with the document review, donor country informants spoke with more certainty about programmes’ impact on domestic research landscapes than on ODA-receiving countries, and interviews overall did not yield robust evidence of concrete development outcomes.

When describing impact on donor country research landscapes, interviewees echoed many of the document review findings. For example, they mentioned programmes’ role in generating and sustaining interest in global health and development research and increasing research institutions’ investment in this field (ODADC interviewees 1–7). As one interviewee noted, “The people that are working on these topics are partly dependent on these types of programmes for their research. So I would say these programmes also help to build and sustain the research infrastructure on, say, inclusive global development and global issues” (ODADC interviewee 6). Programmes were also described as supporting the development of partnerships between donor and recipient country researchers (ODADC interviewees 1–7) that, according to one interviewee, “we wouldn’t have had without these funds” (ODADC interviewee 3).

Interviewees from ODA-receiving countries noted that international funding for research is welcome and that a key pathway to impact in their countries involves capacity-building (ODARC interviewees 1–3). For example, one interviewee observed that collaboration “increases our capacity in science” and gives researchers “an opportunity to actually grow their research” (ODARC interviewee 1). Another interviewee described the results of a development research funding programme as “very positive in that the partnership has actually contributed to [the] capacity of researchers, especially in fields that are new, technologies that are new, and this has contributed to improving our capabilities in specific areas” (ODARC interviewee 3). However, as described in more detail below (theme 4), capacity-building was typically not a direct objective of the programmes we analysed.

A few interviewees identified projects or research areas that had produced applied impact (ODADC interviewees 3, 6, 8; ODARC interviewee 1). Two interviewees mentioned examples of impact in the health field in particular. One noted success in the “implementation of new vaccines” and “[d]evelopment of new diagnostic methods” (ODARC interviewee 1), while another described several health-related projects that had generated concrete results (ODADC interviewee 8). Some donor country interviewees also described research as contributing to the evidence base for development policies or activities (ODADC interviewees 1, 4, 5, 6, 7, 8). As one informant explained,

NGOs [nongovernmental organizations] active in development cooperation that have mandates funded by ODA… base very much on research, on state-of-the-art of knowledge… Some of them implement projects that are very accurate to be implemented in today’s complex systems... And I think that this kind of research programme that we are implementing has the advantage to also contribute to a transformation within the science system. Because with the push from ODA, our researchers are also asked to refer to things that are relevant for development or to frameworks like the 2030 Agenda. (ODADC interviewee 5)

Another interviewee similarly noted that “an important aspect of achieving the [SDGs] is… you need research and you need involvement from the universities and research institutes because… there’s this cross-sectoral approach in them where you need to sort of balance different aspects of development against each other” (ODADC interviewee 8).

At the same time, interviewees commonly described more indirect and/or long-term pathways to development impact and did not express certainty that programmes achieved these outcomes (ODADC interviewees 1, 2, 3, 4, 6, 7). One interviewee noted that funded research is expected to produce different “scalable” outcomes:

So there will be some shorter-term, smaller impacts as a result of that project. And we've got early evidence already for some, and yet there are also, potentially, quite longer-term transformative impacts which will be harder to track back to the research and may not happen for a number of years… I guess it's easier to maybe think of what is the right balance, and are we comfortable in terms of our balance of risk across the different projects. (ODADC interviewee 3)

Informants also explained that the long timelines and uncertain outcomes of research precluded decisive statements on impact or comparisons with the work of development agencies. As one interviewee noted,

I think they’re trying to mostly do different things… It’s a much longer-term gain that we’re hoping will be effective… [T]he impacts of research could be long-term, like 10 years, more… We have clear requirements for demonstrating how this research will impact in different ways through policy or other aspects of society. But you never know, when you’re funding research, how that will happen or if it will happen as planned. (ODADC interviewee 2)

An informant from an ODA-receiving country similarly emphasized that the results of development research programmes are not always clear: “[S]o much research is being done in [ODA-receiving country name], so much. But if we really want to take stock of, okay, how much have we done, what have we invested, what are the outputs, what’s the impact, it’s very difficult to get” (ODARC interviewee 2).

The difficulty in quantifying impact can also be a political challenge in donor countries. As one informant explained:

We do serve multiple goals, and we want to have direct impact in the developing countries where we work. We want also, with our collaboration we want to contribute to…academic capacity-building in those countries. But we also want to provide input for policy-makers here in [donor country name] working on development issues… [R]esearchers, and also our local partners, do think that we're very effective, or that the research we fund is very effective in generating impact locally and strengthening academic capacity locally. We do have trouble, it is difficult to also prove this impact to our policy-makers in [donor country name]. (ODADC interviewee 6)

The interviewee also noted the difficulty in sustaining political interest in research programmes with long timelines for impact (ODADC interviewee 6).

Interviewees identified a number of strategies to translate intermediate outcomes and outputs into end-stage impacts. As described below (themes 4 and 5), informants from ODA-receiving countries highlighted that for their countries to benefit from such research, programmes should emphasize capacity-building and application of new technologies (ODARC interviewees 1–3). Interviewees from ODA donor and recipient countries also noted the importance of structured mechanisms for anticipating and evaluating impact (ODADC interviewees 4, 5, 6; ODARC interviewees 2, 3). One informant stressed that “even without the partnership with a foreign country or a donor, we have certain parameters we expect to be satisfied by the researcher before we can actually issue funds. One of them is actually socioeconomic impact” (ODARC interviewee 3). Another informant noted researchers’ responsibility to demonstrate an impact to the communities on which their research focused and from which they extracted data (ODARC interviewee 2). A donor country interviewee described their programme using “instruments like theories of change and impact pathways as tools for researchers to really systematically think about the output and the outcomes and the impact of research” (ODADC interviewee 6). An interviewee whose programme had ongoing monitoring mechanisms emphasized the importance of being “an engaged funder, an interested funder, to really follow up on what is happening in these projects” (ODADC interviewee 5).

Finally, interviewees noted the importance of “getting this kind of research [to] policy-makers” (ODADC interviewees 1, 2 [quoted], 4, 5, 6, 7; ODARC interviewee 2). One emphasized that for programmes to “add value to ODA and other forms of development cooperation… what is necessary is that researchers are establishing dialogues and exchanges with development cooperation actors and also of course with civil society actors” (ODADC interviewee 5). Another described efforts to “creat[e] more opportunities for intermediary knowledge utilization, to have researchers interact throughout the process, also with people from the ministry, instead of just sending their results after 5 years” (ODADC interviewee 6).

#### 2: Delivering programmes through and with research funding bodies can increase effectiveness and efficiency

Several of the end-stage impacts identified in Fig. 1 rely on generating high-quality research by funding well-designed and well-coordinated projects. Donor country interviewees identified programmes’ operational delivery through national research funders as an institutional design feature that increased project quality, the prestige of development research, and/or the range of disciplines and research councils expressing interest in programmes (ODADC interviewees 4, 5, 8).^7^ According to one interviewee, in comparison with a previous model, “more research[ers] from several disciplines that were previously not engaged that much in research for development had access to this scheme and are actually successfully implementing projects” (ODADC interviewee 5). Another research funder was able to increase research quality over time through its access to scientific expertise: “One of the reasons that the quality has increased in the last years is that researchers have applied again and again and they’ve improved their applications based on the feedback they’ve gotten from us and from these expert panels” (ODADC interviewee 8).

An interviewee from an ODA-receiving country similarly identified gains in efficiency from working with existing research funders in the country where research is conducted, which helps to avoid redundancy and ensure alignment with local priorities (ODARC interviewee 2). The need for coordination among funding agencies was also raised by a donor country interviewee, who said that “the main thing that’s come out of a lot of the interactions or workshops I’ve had with other funders or people we fund… [is] how we, as funders, can work better together to make it better for researchers, not…trying to do similar topics at the same time that will drain that particular research community” (ODADC interviewee 2).

#### 3: Partnerships are more effective when equity is built into programme and funding rules

Each programme in this evaluation aimed to increase research partnerships between ODA donor and recipient countries. Interviewees from ODA-receiving countries considered the involvement of researchers and institutions from their countries as crucial for global health and development research to have a local impact (ODARC interviewees 1–3). As one interviewee noted, “the more participation that you have from the receiving countries, the better it’s going to be, always. Because you’re always going to be able to actually approach the question with a little bit more detail and understanding of these countries” (ODARC interviewee 1).

**Fig. 2:**
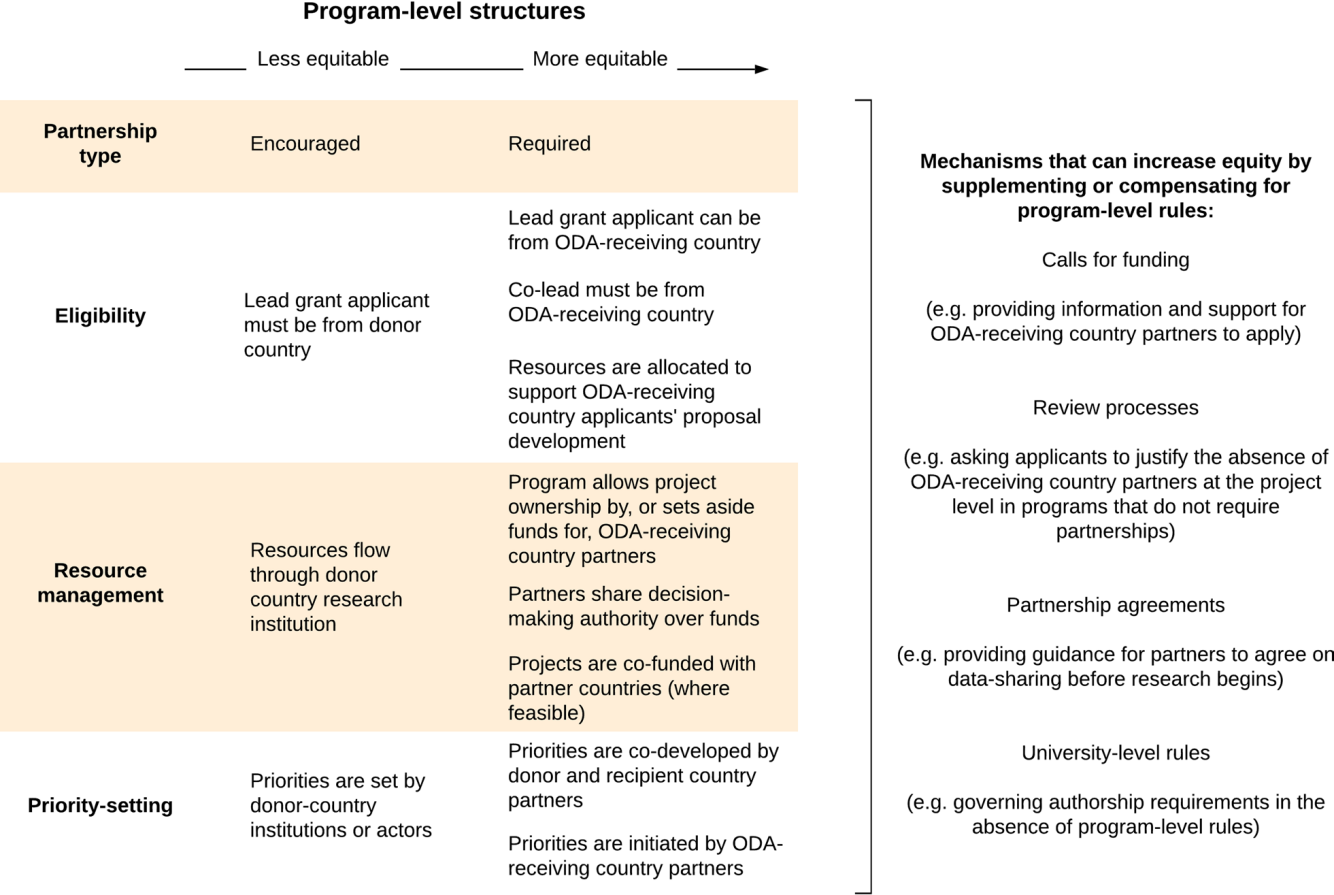
Structures and mechanisms that influence equitable partnerships between researchers in ODA donor and recipient countries (derived from interview and document data)

There was a common recognition among interviewees that the equitability of North–South partnerships is shaped by programme structures, funding rules and application processes (Fig. 2) (ODADC interviewees 1, 2, 3, 4, 5, 7; ODARC interviewees 1, 3). For example, one interviewee described the benefits of building equity into programme structures:

[O]ften, the grant is awarded to one organization. For the majority of cases, that organization, at least at the moment, is in [donor country name]. The risk there is that because they are the primary grant holder, that there could be an inequitability in terms of the way that they distribute funds to partners. So we have to be a lot more careful around enforcing and encouraging equitable approaches in that context [in comparison to programmes where these aspects are built into the programme framework]. (ODADC interviewee 3)

Interviewees from ODA donor and recipient countries identified several elements that can increase the equity and effectiveness of project partnerships, including co-funding and co-development arrangements; measures that allow (or require) project funding to be allocated to researchers in partner countries; provisions giving partners management and decision-making authority; measures that enable researchers from ODA-receiving countries to be primary applicants; and financial support for researchers from ODA-receiving countries to apply for funding and develop proposals together with donor country partners (ODADC interviewees 2, 3, 5, 6; ODARC interviewees 1–3).

Interviewees from ODA-receiving countries expressed strong support for programmes that are co-developed by relevant actors in the donor and recipient countries (ODARC interviewees 1–3). One interviewee said of such an arrangement, that “through collaborative research, meaning from the conceptualization stage, researchers were able to work together to develop a common protocol, up to the stage of implementation. Our researchers definitely learned a lot, and there was a lot of exchange of information and learning between the two sides” (ODARC interviewee 3). Another interviewee similarly commented that the co-development model “makes a big difference in terms of how we actually interact with the researchers in [the donor country] and how our researchers can also co-develop programmes” (ODARC interviewee 1). However, interviewees also observed that co-funding and co-development models require institutional capacity and political support in ODA-receiving countries (ODADC interviewee 2; ODARC interviewees 1, 2).

Donor country interviewees described several ways through which partnerships are regulated, including programme-level rules, integrating concerns with equitability into call requirements and review processes, and relying on delegated mechanisms such as partnership agreements and the rules established by grantees’ universities (ODADC interviewees 1–8). In some cases, call-level or delegated measures were in addition to programme-level requirements; in others, they partially compensated for weaker programme rules. One informant whose programme can only send funding to donor country institutions explained that the grant review panel is tasked with assessing the equitability of potential partnerships, since the programme itself cannot provide the institutional safeguard of granting funds directly to partner countries (ODADC interviewee 4). Another interviewee explained that expert review panels “look very deeply into the partnerships and how they are described in the applications” (ODADC interviewee 7).

After funding is granted, partnership details are typically managed through researchers’ universities and partnership agreements. Again, these mechanisms can be in addition to programme rules or compensatory where built-in requirements are lacking. As one interviewee explained, research partnerships require “a certain formality… So next to the [grant rules]… [w]e ask from all projects to develop a project agreement together and we provide a template… It is not a legally binding document, but it’s a basis for them to start a project together and also to get back to when something happens” (ODADC interviewee 5). An ODA-receiving country informant also emphasized the importance of partnership agreements. They noted several potential problems, such as local researchers “just being data collectors for others” and not being included as authors on publications, and stated that when initiating “a collaborative research project, you have a certain obligation consisting that beforehand you have that kind of terms of reference” about partnership roles (ODARC interviewee 2).

Several programmes had, or were developing, formal rules involving data-sharing and/or open-access publishing (ODADC interviewees 3, 4, 5, 6, 7). One interviewee explained that “ultimately, it’s up to organizations to agree the details around data-sharing and [intellectual property] and things like that. But we do have a framework for data best practice… [A]ll the research that we fund, eventually, the data should be accessible to everyone” (ODADC interviewee 3). Another noted that these issues, which had originally been left to project agreements, were becoming more institutionalized (ODADC interviewee 5).

#### 4: Capacity-building is often donor country-focused and happens more indirectly in partner countries

Multiple donor country interviewees described capacity-building in ODA-receiving countries as a secondary goal, limited aim or welcome by-product of collaboration, rather than a primary objective of their programmes (ODADC interviewees 1, 4, 5, 8). Although they are well suited to build capacity domestically (theme 2), some interviewees suggested that donor country research councils that fund time-limited research projects may be less equipped to build capacity in ODA-receiving countries, whereas national development agencies or other delivery partners may be better suited to this task (ODADC interviewees 1, 2, 8). An informant described the challenge of building capacity through smaller programmes at domestically oriented funding agencies:

[T]here was a discussion… [whether] this actually is feasible for a research programme. To sort of build research capacity and institutions in [ODA-receiving] countries...because research programmes, they fund research projects that sort of have a limited amount of years…and after the project period is over, what happens to those partnerships if there… aren't any more funds? And if they don't get a new project? So… now it's more on a strengthening capacity… [I]t's not our aim to build something from scratch, but rather build upon what is already there… [O]ur capacity's more on developing methods together, developing new research projects, but using the people that are already in the [ODA-receiving] countries. (ODADC interviewee 8)

In several programmes, capacity-building took place at the individual level through skill development and transfer during the collaborative process or via training opportunities for junior researchers from ODA-receiving countries (ODADC interviewees 1, 4, 5, 8). One interviewee noted that capacity-building is very expensive and is not a main focus of their programme, although the programme does support “skills development”, which is “also kind of capacity-building” (ODADC interviewee 5). Another informant described their programme’s main goal as strengthening donor country knowledge on development issues and explained that enhancing development research competence among donor country institutions will enable them to “include partners themselves, and then strengthen not only their own competence through that, but also the competence in the recipient country” (ODADC interviewee 1). The interviewee also noted that trainees from ODA-receiving countries can receive funding through programme projects (ODADC interviewee 1). The latter approach was mentioned as beneficial by informants from ODA-receiving countries when “the donor country actually receives people from the [partner country…]. That creates a movement of people. That’s fundamental for the process of generating more and more knowledge, capacity-building, and can also increase the capacity to discuss science” (ODARC interviewee 1).

In cases where capacity-building was an explicit goal at either the programme or call level, interviewees mentioned specific measures that were in place to ensure co-leadership and/or equitable funding access (ODADC interviewees 2, 3). Speaking about a call for funding that had capacity-building in developing countries as a primary objective, one interviewee described the corresponding decision to allow funding to cover both direct costs and more indirect institutional costs in partner countries (ODADC interviewee 3). Funded projects were also required to have a plan for sustainability, because “[t]here’s no point in having the capacity-building built up and then forgotten about after 4 years. That was built in there, and we asked our review panel to assess the potential sustainability based on the case that they made” (ODADC interviewee 3). Another interviewee similarly highlighted the value of targeted capacity-building:

[T]here are other projects that have happened and they have combined research and capacity development. Those have been very very beneficial in the sense that they actually try to address the problem better by using research. At the same time, they do incorporate the capacity-building for the developing country members in the sense that, if we were all doing that at a certain time… the country would have had enough capacity to be able to at least initiate some of the studies themselves. (ODARC interviewee 2)

They contrasted this with a model where “the actual conceptualization has been done from outside, the data is just being collected here, and then it goes outside without the benefit of analysis and understanding and capacity-building” (ODARC interviewee 2). Another informant spoke positively of a programme that had capacity development as a goal and in which there was a deliberate focus on identifying the priority areas for research investment in the ODA-receiving country and “matching from the [donor country’s] side, who are the researchers [that] could actually work with us on that particular area” (ODARC interviewee 3).

#### 5: Programme priority-setting processes are often top-down and donor-led, but call- and project-level priorities are more flexible and opportunities exist to pursue mutual goals

Several donor country interviewees described programme-level priority areas being set by or developed with national foreign affairs ministries and/or development agencies (ODADC interviewees 1, 2, 3, 5, 6, 8). Interviewees explained that programme “thematic areas correspond fully with the development priorities of the [national development agency]” (ODADC interviewee 5) and that “the general themes…have to align with the ministry” (ODADC interviewee 6). In a third programme, “the initial priority-setting came from…a government-led process” and the programme “priorities have sort of been set as the international trend and international development within this field…has changed” (ODADC interviewee 8). Other donor country interviewees also mentioned global concerns and international agendas when describing programme goals and described broad priority areas, including topics like global health, education, inclusive development, climate and environment, and sustainability (ODADC interviewees 1, 3, 4, 5, 6, 8).

Within these broad areas, funding agencies usually had flexibility to design more specific or flexible calls (ODADC interviewees 2, 3, 5, 6, 8). One interviewee explained that as a “governmental research funder, we are dependent on getting funds from the ministry. So it’s been a dialogue between us and the ministry on what type of priorities we should have for this programme” (ODADC interviewee 8). They went on to clarify:

[A]ll those formulations on what the different topics should be, it's not something that we received from the ministry, that's something that's been developed here at the [research funder], but also with our programme board… [The] priorities are linked to funding of course, but… the demands that come from the ministries... They're not very specific… it's up to us to sort of determine the details and the level of the priorities. (ODADC interviewee 8)

Interviewees from ODA-receiving countries stressed the need for consultation with local partners in deciding specific priorities (ODARC interviewees 1–3). One identified the risk of a “lack of understanding sometimes the real priority of the country” when programmes are initiated externally, and added that “the development of priorities together with the countries is a very important issue” (ODARC interviewee 1). This was consistent with another informant’s statement that “what we would like to happen is that any assistance for that matter should really be only for the areas that we think are a priority in [ODA-receiving country name]” (ODARC interviewee 3). A third informant noted that “research projects that have come from developed countries, they all differ… [S]ometimes even the topic or the type of development research they want to do comes from outside… If we are aiming at this development research, we really need to address the problems that are within the context of that country where the research is going to be conducted” (ODARC interviewee 2).

Interviewees noted that a focus on local priorities does not preclude investing in issues of global relevance or producing results that benefit both ODA donor and recipient countries (ODARC interviewees 1–3). For example, one interviewee who emphasized the need to respect in-country priorities also observed, regarding donor-initiated research, that “in the past, a lot of people said, this is like… people from outside trying to dominate what you do in the country, but I do not think that people see it that way anymore… because science is a kind of worldwide kind of activity” (ODARC interviewee 1). Another interviewee highlighted the alignment of their country’s national priorities with the SDGs and other global agendas and additionally mentioned that climate change has exacerbated the lack of widespread in-country uptake of certain technologies related to food production (ODARC interviewee 2).

Interviewees additionally observed that investments in scientific development could maximize their benefit to developing countries by considering the type of research and technology most relevant to and necessary in these contexts (ODARC interviewees 1–3). One interviewee observed, “I do understand that it should be mutually beneficial, but I think we can match in terms of common areas of priority, that the two countries can actually invest, so that the resource will be more impactful” (ODARC interviewee 3). They emphasized the need for investments in technology that benefit both sides:

For example, in a particular research area, is it something that’s not yet available in [ODA-receiving country name]? So if it’s not available in [ODA-receiving country name], then there’s enough reason for us to pursue that research with the partner country. And hopefully, learning the technology will then be transferred to [ODA-receiving country name]. (ODARC interviewee 3)

Another informant noted the importance in health research of having in-country clinical trials “from phase 1 up” rather than only later stages that are not as focused on “scientific development” (ODARC interviewee 1). A third informant observed that “applied technologies [in a particular sector] have hardly reached” their country and added that “we would like to have such research in the country that would actually demonstrate the impact of application of technologies to improve the average [person’s] life” (ODARC interviewee 2). The interviewee observed that their country had much to contribute to global knowledge development but also needed assistance translating that knowledge into locally beneficial technologies and applications:

[W]e have so much indigenous knowledge, but uptake or formalization is always a challenge. So the type of project that would actually benefit both sides would rest on that, and I’m sure a lot can be learned. We have indigenous knowledge, we have abundant biodiversity to share, but that won’t make any meaning if we cannot partner with those who are able to make use of such a rich environment. So I think we both need each other…and we have actually identified research as one of the main ways to bring about sustainable development. (ODARC interviewee 2)

A donor country interviewee similarly highlighted the role of research in contributing to core capacity in developing countries and finding cost-effective ways to deliver GPGs:

[T]he amount of ODA that’s available wouldn't really cover the needs of… a health sector in a developing country, or several developing countries… So I think it's one of the important things to figure out, while trying to achieve the [SDGs], is how we can enable developing countries on financing their own health sectors and finding their own ways of providing healthcare services to their population… And it's important to figure out the cost-effectiveness of the different interventions… Even though you have a really good drug that could save a lot of people, but if that drug is unbelievably expensive, then there won't be that many countries that could use it, so I think that's an important factor of research. (ODADC interviewee 8)

The Newton Fund, which has a built-in structure for co-development between the donor and partner countries, provides the clearest programme-level example of a process for identifying common priorities.^8^ Other programmes included or were considering consultation processes with ODA-receiving country researchers or stakeholders at the call and/or project level (ODADC interviewees 3, 4, 7). One interviewee described an intensive process for setting priorities that included a strategic body with “developing country and user representation” (ODADC interviewee 3). The interviewee noted that although the process differed across thematic areas, “the principles are there in terms of engaging in the right kind of level including the developing country researchers as well as…user or stakeholder organizations” (ODADC interviewee 3). Another interviewee identified as a strength of their programme a project-level emphasis on working with “national authorities in order to make the research as relevant as possible for the context in which they are doing the research” (ODADC interviewee 7).

## Discussion

### Principal findings

This article assessed the impact and institutional design of ODA-funded granting mechanisms for global health and development research that is initiated in donor countries. Based on the assessment of relevant documents as well as interviews, we found that ODA-funded research programmes constitute a promising approach to producing valuable knowledge of relevance to ODA-receiving countries when they:

- focus on local priorities and absorptive capacity,
- translate GPGs-relevant research into technologies that are appropriate for developing-country contexts,
- include overall strategies and ongoing monitoring mechanisms for ensuring ODA relevance and development impact,
- build in structures for equitable partnerships,
- strengthen individual and, if possible, institutional capacity in ODA-receiving countries, and
- ensure opportunities for knowledge mobilization.

Delivering programmes through research funding bodies with established expertise can also contribute to effectiveness and efficiency by enhancing research quality and coordination with other funding bodies. However, in the absence of concrete evidence of development impact, increases in development research spending should be additional to rather than a substitute for direct investments in ODA-receiving countries that have demonstrated effectiveness.

### Implications for policy

#### Recommendation 1: Consider ODA-funded global health and development research programmes as a tool to strengthen the knowledge base, and collaborative capacity, in this field

Donor countries should consider the potential for ODA-funded research programmes to address gaps in their institutional frameworks for addressing development goals and global challenges. While this article shows that programmes’ impact on development outcomes depends on careful design and implementation, overall our analysis found the model to provide promising pathways to generating collaborative research that can inform global health and development policy and practice, develop broadly applicable technologies, and strengthen capacity in ODA-receiving countries. Countries that choose to adopt this approach should consult the wealth of experience from existing programmes in ODA donor countries, and from recipient countries where these have been implemented (Table 6).

**Table 6:** Recommendations based on overarching findings from interview data and document review

1. Consider ODA-funded global health and development research programmes as a tool to strengthen the knowledge base, and collaborative capacity, in this field
2. Focus on addressing local research priorities and strengthening absorptive capacity
3. Ensure that research oriented towards GPGs addresses the development or adaptation of technologies that are appropriate, acceptable, affordable and based on needs in developing-country contexts
4. Build in coherent strategies and monitoring and evaluation mechanisms to maximize and clarify ODA-relevant outcomes
5. Make equitable partnerships part of the programme structure and add supplementary provisions at the call and project levels that make partnerships even more equitable
6. Clarify when capacity-building is an explicit goal of ODA-funded research programmes and include specific provisions for this purpose
7. Ensure opportunities for engagement between researchers, policy-makers and development actors to deepen the evidence base for development activities
8. Consider leveraging the expertise of existing research funders in ODA donor and recipient countries to deliver ODA-funded research programmes

#### Recommendation 2: Focus on addressing local research priorities and strengthening absorptive capacity

Our document synthesis and interviews highlighted the need to balance investments in research on issues of immediate and localized development impact with longer-term projects and those with potentially scalable and transformative results. In line with the Paris Declaration and Accra Agenda for Action, ODA-funded research in both categories should address ODA-receiving country priorities. There are several ways to achieve this through research, including by addressing issues specific to a particular country or group of countries; by conducting research that strengthens in-country capacity to absorb and implement new knowledge and technologies (e.g. through health systems research, phase 1 trials); and, as discussed below (recommendation 3), by ensuring that research on GPGs results in technologies that are useful, affordable and accessible in developing-country contexts. In all cases, consulting recipient countries at the earliest stage and highest programmatic level possible is crucial. This can be facilitated through co-development at the government level, representation on boards that set call-level themes, and working with researchers, institutions and other relevant actors at the project level in contexts where the research occurs. As donor countries consider aligning ODA allocations with STI investments to address global challenges, they should keep in mind the complementarity of country-specific investments and GPGs provision, and consider making investments in GPGs additional to ODA spending on health, education and other sectors that are critical for developing countries to benefit from new knowledge and technologies (e.g. [29, 31, 57, 58]).

#### Recommendation 3: Ensure that research oriented towards GPGs addresses the development or adaptation of technologies that are appropriate, acceptable, affordable and based on needs in developing-country contexts

In addition to ensuring continued investments in national systems that are key to delivering GPGs, ODA-funded research that addresses global concerns should be translated into technologies that are appropriate and accessible in developing-country contexts. As interviews confirmed, research oriented to GPGs such as medical interventions, climate stability, clean energy and food security can align strongly with the priorities of ODA-receiving countries. However, investments in these areas benefit these countries most when attention is paid to the forms of technology that are needed in countries at different stages of development. To ensure that GPGs-oriented research does not displace other research or implementation programmes, it is important to have better information on donor countries' investments in GPGs both within and outside their ODA budget envelope, since such investments benefit both developed and developing countries and should come from domestically oriented and ODA budgets.

#### Recommendation 4: Build in coherent strategies and monitoring and evaluation mechanisms to maximize and clarify ODA-relevant outcomes

The multiple goals and pathways to impact of ODA-funded global health and development research programmes involve different time frames and criteria for evaluation. This highlights the need for coherent programme strategies that establish a theory of change from the outset, as well as ongoing monitoring to ensure—and make evident—their impacts on the ground. Although monitoring and evaluation activities can be resource-intensive and involve trade-offs in programme budgets, elucidating development outcomes is critical to ensure that ODA spending meets the needs of ODA-eligible countries and is compliant with its stated goals throughout the project. It is also key to fulfilling researchers’ responsibilities to the communities they study and to securing political support in donor countries. While orienting research to GPGs can be politically attractive due to the possibility of mutual benefits, the longer time frames of such projects can also make it harder for politicians to see the value of sustaining or increasing investment. Any prospect for scaling up this funding approach to make a substantial contribution to global challenges is therefore likely to be strengthened by demonstrating concrete impact. Where resources for monitoring and evaluation are limited, outcome-tracking could be improved by requiring applicants to include theories of change and impact plans in proposals and to report on these during the granting period, as well as by requesting updates on development impact a couple of years after the funding period has concluded and combining this with data from digital sources.

#### Recommendation 5: Make equitable partnerships part of the programme structure and add supplementary provisions at the call and project levels that make partnerships even more equitable

ODA-funded research programmes aim to produce knowledge relevant to the development of ODA-eligible countries. Our findings concur with a vast literature detailing the importance of having researchers from these countries involved in producing that knowledge. This article identified several ways to incorporate equity at the programme level, including enabling developing country researchers to lead projects and have equal access to/control over funding. We also identified mechanisms that supplement these rules or partially compensate for their absence. In light of the power imbalances that can exist between researchers from ODA donor and recipient countries, a structured approach strengthened by supplementary mechanisms is warranted. Attention to partner-country context is also critical. Co-funding arrangements should be considered with care in light of the potential exclusion of countries with fewer research resources and due to concern around spending ODA funds primarily in the donor country in cases where partner countries fund the portion of research conducted domestically.

#### Recommendation 6: Clarify when capacity-building is an explicit goal of ODA-funded research programmes and include specific provisions for this purpose

One way in which global health and development research programmes can have a more direct impact in ODA-receiving countries, address structural imbalances between research communities in the Global North and South, and strengthen the basis for more equitable research collaboration involves investing in research capacity in partner countries at both the individual and institutional levels. Programmes are more likely to achieve this when capacity-building is a central goal that is accompanied by targeted financial and/or structural elements, as is done by several programmes run by development agencies or delivery partners with more organizational capacity to accomplish this objective. Donor countries should carefully consider enhancing the capacity-strengthening aspects of global health and development research programmes to the extent possible within institutional and contextual constraints, including by consulting with ODA-receiving countries about existing gaps in capacity and focusing collaborations on those areas; allowing project ownership and fund management by ODA-receiving country institutions; ensuring the co-development of projects from proposal to implementation to maximize skills transfer; enabling junior researchers from ODA-receiving countries to access training through the programme; and at the call level, asking applicants to include plans for capacity-building legacy after the funding period has ended. Where it is not feasible to build long-term capacity through programmes run by domestically oriented research councils, countries should consider establishing such programmes through agencies with more resources available for this purpose.

#### Recommendation 7: Ensure opportunities for engagement between researchers, policy-makers and development actors to deepen the evidence base for development activities

One of the pathways to impact for global health and development research programmes involves providing a stronger evidence base for the use of other ODA funds through exchanges with policy and development actors in donor and recipient countries. This is particularly important in light of remaining concerns about overall aid effectiveness (e.g. [59, 60]). This article identified several programmatic elements intended to achieve this impact, including requiring a portion of grant budgets to be spent on dissemination activities; holding periodic meetings between researchers and policy-makers; and facilitating more regular dialogues between project partners and societal and policy actors.

#### Recommendation 8: Consider leveraging the expertise of existing research funders in ODA donor and recipient countries to deliver ODA-funded research programmes

The experience of research funding agencies in ODA donor and recipient countries was identified as beneficial to delivering high-quality and well-coordinated development research programmes. Delivering such programmes through donor country institutions with a strong reputation for peer-reviewed granting processes was seen as increasing the prestige of development research and promoting its integration within the broader research community—although the strengths of research funders in fostering high-quality research must be paired with more rigorous procedures for ensuring the ODA relevance of funded projects. The inclusion of such funding programmes within established research funders may also allow for creating combined programmes that are both ODA and non-ODA (domestically) financed. This may be a particularly useful approach in the era of the SDGs, where goals are universal and where the technologies, interventions, practices and policies needed for change may be relevant across contexts. Communication among these bodies is also crucial to increase learning and efficiency and avoid duplication and fragmentation. Coordinating with (or helping to strengthen) research organizations in ODA-receiving countries is also important to ensure the appropriateness and context sensitivity of research and its alignment with local priorities.

### Strengths and limitations of this study

There are three main strengths and two main weaknesses of this study. The first strength involves our combination of document and interview data to triangulate our findings and deepen our understanding of the programmes we evaluated. Second, we interviewed senior individuals from each donor country programme. The interviewees were in a position to evaluate the programmes broadly while providing specific details and examples. Third, we incorporated perspectives from senior individuals at research funders in ODA-receiving countries that represent a range of income levels and regional contexts. Because these individuals were highly knowledgeable about development research and partnerships based on their involvement in numerous international projects, we gained an understanding of the specific issues that matter for impact in ODA-receiving country contexts.

One limitation of this project was that we spoke to a small number of individuals in ODA-receiving and donor countries, and therefore did not hear a variety of perspectives from each context. We believe that reviewing programme documents compensated somewhat for this limitation in the donor country cases by providing additional sources of information, though we were of course limited to documents that were publicly accessible. Furthermore, the interviewees from ODA-receiving country funders were privy to the feedback of many researchers and institutions and were therefore able to base their responses on broad knowledge of their contexts. A second limitation was that the interviewees all worked for granting agencies that may be implicated in decisions about global health and development research funding. However, the candour of the interviewees suggests that they have a strong motivation to ensure that these programmes maximize their impact through effective design.

### Future research directions

In light of the paucity of concrete evidence concerning the direct impact of the evaluated programmes on global health and development outcomes, further studies are needed to elucidate the effects of the funded research across different time frames. Future research might also compare different models of delivery, including programmes operated by externally oriented institutions (such as development research institutions and development agencies) compared with more domestically oriented research funders, to better understand the impact of institutional design on programme outcomes.

## Conclusions

Global health and development research programmes and partnerships have an important role to play in achieving the SDGs and addressing global challenges like the COVID-19 pandemic response and recovery. However, work remains to ensure that ODA-funded research that is initiated in donor countries maximizes its relevance and outcomes for ODA-receiving countries. Through an analysis of seven comparable programmes, this article provided a novel assessment of this funding approach and highlighted the key role of institutional design when it comes to setting priorities, building partnerships, strengthening capacity, assessing and coordinating projects, and strategizing and monitoring impact. With limited resources available and serious challenges ahead, it is critical for governments to consider the potential of ODA-funded research in light of the experiences of existing programmes.

## Supplementary Information

**Additional file 1: Appendix S1.** Donor country interview guide. **Appendix S2.** ODA-receiving country interview guide.

**Additional file 2: Appendix S3.** List of documents and website descriptions analysed.

## Data Availability

A list of documents included in the analysis is available as Additional file 2.

## Abbreviations

COHRED: Council on Health Research for Development
DAC: Development Assistance Committee
FIOCRUZ: Oswaldo Cruz Foundation
GCRF: Global Challenges Research Fund (United Kingdom)
GLOBVAC: Global Health and Vaccination Research (Norway)
GPGs: Global public goods
LMICs: Low- and middle-income countries
NORGLOBAL: Norway—Global Partner
WOTRO: Science for Global Development (Netherlands)
ODA: Official development assistance
ODADC: ODA donor country interviewee
ODARC: ODA-receiving country interviewee
r4d: Program for Research on Global Issues for Development (Switzerland)
SDGs: Sustainable Development Goals
STI: Science, technology and innovation

## Footnotes

1 It is unclear whether this is part of the general increase in investment in GPGs from other budgetary sources, since no agreed indicators or statistics on this topic are available.

2 See Additional file 2 for lists of analysed documents.

3 We interviewed eight donor country informants associated with seven programmes. There was an instance where two research funder employees spoke to us about the same programme over the course of one interview.

4 COHRED is a global nonprofit organization that works with governments, research institutions and civil society to strengthen research and innovation capacity in low- and middle-income countries and promote equitable international research collaborations.

5 These WOTRO programmes were the focus of our analysis.

6 Tied aid is aid that must be spent on goods or services from the donor country [55].

7 This was corroborated by our document review. For example, a review of the GCRF noted its strength in “draw[ing] on well-established mechanisms for identifying research excellence and adapt[ing] them to the requirements of official development assistance” [44], and the r4d programme was seen as benefiting from the development agency’s and science foundation’s dual involvement, with the former emphasizing applied research and the latter enhancing the “research-based pedigree and reputation” [45]. At the same time, concerns have been raised in the United Kingdom that research funding organizations’ focus on research excellence has not always been accompanied by rigorous processes for ensuring ODA compliance or assessing development impact [40].

8 This process has improved over time within the Newton Fund, with a collaborative approach to identifying mutual priorities with partner countries more evident in the second funding period than the first [40].
