# Supplementary material for: Evaluating official development assistance-funded granting mechanisms for global health and development research that is initiated in high-income countries
Source: Health Res Policy Syst. 2022 May 16;20:55. doi: 10.1186/s12961-022-00859-6 (PMC9109198; doi:10.1186/s12961-022-00859-6)
Supplement: Supplementary file 1 — Additional file 1: Appendix S1. Donor country interview guide. Appendix S2. ODA-receiving country interview guide. [file 12961_2022_859_MOESM1_ESM.pdf]

Appendix 1: Donor country interview guide

Introduction

This project is an independent initiative led by the Global Strategy Lab, an interdisciplinary research program at York University in Toronto. The evaluation aims to examine the opportunities, challenges, and institutional design considerations associated with granting mechanisms that use Official Development Assistance (ODA) to provide funding for donor-country researchers to initiate research on development issues. These programs typically offer competitive grants to researchers at institutions in the donor country who initiate research projects of relevance to ODA-receiving countries. Fieldwork often takes place in ODA-receiving countries and partnerships with researchers at institutions in these countries may be encouraged or required.

Through this interview, we hope to build on the information we've gathered from our review of documents by confirming information about this mechanism and asking questions about its design and operational context. We aim to develop recommendations for countries that are interested in adopting or improving this funding model to address development challenges. Consequently, our focus is not on evaluating individual programs, but synthesizing what we learn from those programs to evaluate the approach overall.

We will record the interview and transcribe it so that we accurately capture our conversation. When we write up the project results, we may choose to include some quotes from your interview, but we will not link those quotes with your identity.

Confirming Facts

We'd like to start by confirming some information about the program.

1. We're aware that this program is partially funded through ODA. What percentage of the budget is counted as ODA?

[Additional questions in this section were tailored to each specific program]

Institutional Design and Implementation

One of the goals of this project is to help countries that are considering this approach to learn from the experiences of existing programs. We'd like to understand how this program is designed, how it works in practice, and any elements that have been identified, or that you would recommend, to strengthen the program.

2. From your perspective, has this program changed the research landscape in your country? If so, how?

3. In your opinion, how does this program's effectiveness compare to that of more direct ODA allocations that fund programs and services in recipient countries?

4. In your opinion, is the program a cost-effective way to make progress on development goals?

5. From your perspective, might there have been any unintended negative consequences of this program?

6. To what extent has this funding approach promoted coordination between the research community and policymakers or development agencies in your country?

7. How does the program design promote equitable research partnerships and sustainable research capacity-building in ODA-receiving countries?

8. To your knowledge, what kind of political support or opposition has the program encountered in your country?

Operational Context

Now that we've discussed the program's design, we'd like to identify operational considerations to inform countries that might be interested in adopting this mechanism.

9. How are priorities set for this program and who is involved in the priority-setting process?

10. Can you describe the operational resources required to manage and deliver this program?

Final Thoughts

11. Having reflected on the design and operation of your program, do you have any advice for countries considering this funding approach on how they might design, develop, or implement similar programs that provide ODA funding to donor country researchers?

Appendix 2: ODA-receiving country interview guide

Introduction

This project is an independent initiative being led by the Global Strategy Lab, an interdisciplinary research program at York University in Toronto. The evaluation aims to examine the opportunities, challenges, and institutional design considerations associated with granting mechanisms that use Official Development Assistance (ODA) to provide funding for donor-country researchers to initiate research on development issues. These programs typically offer competitive grants to researchers at institutions in the donor country who initiate research projects of relevance to ODA-receiving countries. Fieldwork often takes place in ODA-receiving countries and partnerships with researchers at institutions in these countries may be encouraged or required.

Through this interview, we hope to understand your perspective on whether this funding approach is an appropriate tool for addressing your country's development and research priorities and how these programs' design contributes to, inhibits, or might improve their effectiveness. Our goal is to develop recommendations for countries that are interested in adopting or improving this approach to address development challenges. Consequently, our focus is not on evaluating individual programs, but on evaluating the approach overall.

We will record the interview and transcribe it so that we accurately capture our conversation. When we write up the project results, we plan to mention which agencies we spoke with and we may choose to include quotes from your interview, but we will not directly attribute any quotes to you or your institution.

Evaluating the Funding Approach

1. From your perspective, have these types of development research funding programs instituted in donor countries changed the research landscape domestically in your country? If so, how?

2. In your opinion, how does the effectiveness of this research funding approach compare to that of more direct ODA allocations that fund programs and services in recipient countries?

3. In your opinion, is this approach a cost-effective way to make progress on your country's development goals?

4. From your perspective, might there have been any unintended negative consequences of this approach?

5. From your perspective, does the design of this approach promote equitable research partnerships and sustainable research capacity-building in ODA-receiving countries? If so, how?

6. To your knowledge, how is this ODA research funding mechanism perceived by the broader development and research communities in your country?

Final Thoughts

7. Having reflected on this funding model, what key considerations would you like to see prioritized by donor countries when they design, or consider adopting, this funding approach?
