# Supplementary material for: Evaluating official development assistance-funded granting mechanisms for global health and development research that is initiated in high-income countries
Source: Health Res Policy Syst. 2022 May 16;20:55. doi: 10.1186/s12961-022-00859-6 (PMC9109198; doi:10.1186/s12961-022-00859-6)
Supplement: Supplementary file 2 — Additional file 2: Appendix S3. List of documents and website descriptions analysed. [file 12961_2022_859_MOESM2_ESM.pdf]

Appendix 3: List of Documents and Website Descriptions Analyzed

Science for Global Development (NWO-WOTRO)

1 NWO. WOTRO Science for Global Development. Netherlands Organisation for Scientific Research; No Date.

2 NWO-WOTRO Science for Global Development [Internet]. [cited 2019 May 16]. Retrieved from: https://www.nwo.nl/en/about-nwo/organisation/nwo-domains/wotro

3 NWO. WOTRO 50 Years - Forward Thinking [Internet]. The Hague: Netherlands Organisation for Scientific Research; 2014 Oct [cited 2019 Aug 25]. Retrieved from: https://www.wereldinwoorden.nl/wp-content/uploads/2013/12/WOTRO50YearsForwardThinking.pdf

4 NWO. Science and Development: Mutual Inspiration [Internet]. The Hague: Netherlands Organisation for Scientific Research; 2010 [cited 2019 Jan 3]. Retrieved from: https://www.nwo.nl/en/about-nwo/media/publications/wotro/wotro---strategy-plan-2011---2014.html

5 NWO. Connecting Science and Society [Internet]. The Hague: Netherlands Organisation for Scientific Research; 2018 [cited 2019 Jan 3]. Retrieved from: https://www.nwo.nl/sites/nwo/files/documents/NWO_strategy_2019-2022_Connecting_Science_and_Society.pdf

6 Kusters K, Huijbregts D, Marinovich G. Research for global development [Internet]. The Hague: Netherlands Organisation for Scientific Research; 2012 [cited 2019 Jan 3]. Retrieved from: https://www.researchgate.net/publication/272823105_Research_for_global_development_-_Examples_of_NWO-WOTRO_funded_research

7 NWO. Global Health Policy and Health Systems Research Programme [Internet]. The Hague: Netherlands Organisation for Scientific Research; 2016 Nov [cited 2019 Jan 3]. Retrieved from: https://www.wereldinwoorden.nl/wp-content/uploads/2013/12/Global-Health_FINAL.pdf

8 Leiden University. Assessing bibliometric performance of NWO-WOTRO funded research [Internet]. The Hague: Centre for Science and Technology Studies, Leiden University; 2017 Oct [cited 2019 May 15]. Retrieved from: https://www.nwo.nl/sites/nwo/files/documents/WOTRO%20-%20CWTS%202017.pdf

9 NWO. WOTRO's Regulations: Supplementary WOTRO Funding Conditions. The Hague: Netherlands Organisation for Scientific Research; 2015 Dec.

Global Health and Vaccination Research (GLOBVAC)

10 The Research Council of Norway. About the Programme: Global Health and Vaccination Research (GLOBVAC) [Internet]. 2018 [cited 2018 Nov 16]. Retrieved from: https://www.forskningsradet.no/en/Funding/GLOBVAC/1253991119808/

11 Global Health and Vaccination Research (GLOBVAC) - Work programme 2012–2020 [Internet]. Oslo, Norway: The Research Council of Norway; 2017 [cited 2019 Feb 28]. Retrieved from: https://www.forskningsradet.no/siteassets/programmer/programplaner/globvac-work-programme.pdf

12 Research Council of Norway. GLOBVAC – Research and Capacity Building for Global Health [Internet]. Oslo, Norway: Research Council of Norway; 2009 [cited 2019 Aug 25]. Retrieved from: https://www.forskningsradet.no/om-forskningsradet/publikasjoner/2009/globvac---research-and-capacity-building-for-global-health/

13 Research Council of Norway. GLOBVAC Annual Report 2017. 2017 [cited 2019 Apr 3]; Retrieved from: https://www.forskningsradet.no/servlet/Satellite/?cid=1254035007752&pagename=VedleggPointer&target=_blank

14 de Jongh T, Varnai P, Melin G, Svetachova M, Grudin M, van Barneveld J. Mid-term evaluation of the second programme for Global Health and Vaccination Research (GLOBVAC2) [Internet]. Amsterdam, Netherlands: Technopolis Group; 2016 Feb [cited 2019 Aug 25]. Retrieved from: https://www.technopolis-group.com/wp-content/uploads/2016/03/Final-report_20160219_clean.pdf

Norway – Global Partner (NORGLOBAL)

15 Work programme 2017–2024 [Internet]. Lysaker, Norway: The Research Council of Norway; 2017 [cited 2019 May 13]. Retrieved from: https://www.forskningsradet.no/siteassets/programmer/programplaner/norglobal-work-programme.pdf

16 Annual report 2017 Norway – Global partner (NORGLOBAL-1) (2009-2014) [Internet]. The Research Council of Norway; 2017 [cited 2019 Mar 13]. Retrieved from: https://www.forskningsradet.no/en/system-pages/search-results/?q=norglobal

17 Annual Report 2017 Norway - Global Partner (NORGLOBAL-2) (2016-2023) [Internet]. The Research Council of Norway; 2017 [cited 2019 May 13]. Retrieved from: https://www.forskningsradet.no/en/system-pages/search-results/?q=norglobal

18 Research Council of Norway. Up to NOK 120 million for high quality research on international development [Internet]. Norway - Global Partner. 2019 [cited 2019 Feb 26].

Retrieved from: https://www.forskningsradet.no/en/Funding/NORGLOBAL2/1254037696770&WT.mc_id=prgfor-utl-NORGLOBAL

19 Melber H. Knowledge is power and power affects Knowledge: Challenges for research collaboration in and with Africa. Africa Development. 2015;40(4):21-42–42.

20 Solberg E, Tellmann SM, Aanstad S, Aksnes DW, Ramberg I, Båring P. Pathways to global impact - Tracing the impacts of development research funded by the Research Council of Norway [Internet]. Oslo, Norway: Nordic Institute for Studies in Innovation, Research and Education (NIFU); 2017 [cited 2019 Aug 25]. Report No.: 13. Retrieved from: https://nifu.brage.unit.no/nifu-xmlui/bitstream/handle/11250/2446640/NIFUreport2017-13a.pdf?sequence=4&isAllowed=y

21 Romanow B, Horrigmo AMJ. Ex Post Evaluation of the PovPeace Programme [Internet]. Oxford Research; 2015 Apr [cited 2019 Aug 25]. Retrieved from: https://evalueringsportalen.no/evaluering/ex-post-evaluation-of-the-povpeace-programme-2/POVPEACE%20Final%20report.pdf/@@inline

22 Herman J, Pantuliano S, Sorensen JS. External Review Report of the NORGLOBAL/Humanitarian Policy Research Activity (HUMPOL) of the Research Council of Norway [Internet]. 2016 Apr [cited 2019 Aug 25]. Retrieved from: https://evalueringsportalen.no/evaluering/external-review-report-of-the-norglobal-humanitarian-policy-research-activity-humpol-of-the-research-council-of-norway/HUMPOL.pdf/@@inline

Program for Development Research (Sweden)

1.

23 Strategy for research cooperation and research in development cooperation 2015 – 2021 [Internet]. Stockholm, Sweden: Government Offices of Sweden; 2015 [cited 2019 Mar 13]. Retrieved from: https://www.government.se/49f23e/contentassets/f929d648751e40859c7eb39812dacb01/strategi-for-forskningssamarbete-pdf-for-webb-eng-2.pdf

24 The Committee for Development Research [Internet]. Swedish Research Council. 2018 [cited 2018 Nov 14]. Retrieved from: https://www.vr.se/sidan-kan-inte-hittas.html

25 Brief on Development Research at Swedish Research Council. Swedish Research Council; No Date.

26 Hårsmar M, Fellesson M. Review of Sida's Programme for Development Research [Internet]. Stockholm, Sweden: Swedish International Development Cooperation Agency; 2013 Feb [cited 2019 May 28]. Retrieved from: https://www.sida.se/contentassets/8a2361ad07a24dcea56e224d589352a3/15368.pdf

27 Research project grant: Sustainability and resilience – Tackling consequences of climate and environmental changes [Internet]. Swedish Research Council. 2018 [cited 2019 Mar 13]. Retrieved from: https://www.vr.se/sidan-kan-inte-hittas.html

28 Research project grant - Development Research [Internet]. Swedish Research Council. 2018 [cited 2019 Mar 13]. Retrieved from: https://www.vr.se/sidan-kan-inte-hittas.html

29 Network Grant – Swedish Research Links [Internet]. Swedish Research Council. 2018 [cited 2019 Mar 13]. Retrieved from: https://www.vr.se/sidan-kan-inte-hittas.html

30 Hagfeldt A, Albihn M, Bigsten A, Friberg P, Gill M, Hackman H, et al. The Future of Swedish Research! Overview 2014 Development Research [Internet]. Stockholm, Sweden: Swedish Research Council; 2015 [cited 2019 Mar 13]. Retrieved from: https://www.vr.se/download/18.5f55e5e81618e003b7066fa0/1555426928229/Future-Overview-2014-Development-Research_VR_2015.pdf

Program for Research on Global Issues for Development (r4d)

31 Guidelines for the lifetime management of r4d projects [Internet]. Swiss National Science Foundation; 2015 Sep [cited 2019 Mar 26]. Retrieved from: http://www.r4d.ch/SiteCollectionDocuments/Guidelines%20Lifetime%20Management%20r4d%20projects.pdf

32 Management Principles r4d Social Conflicts [Internet]. Swiss Programme for Research on Global Issues for Development; 2015 May [cited 2019 Mar 27]. Retrieved from: http://www.r4d.ch/SiteCollectionDocuments/SC_ManagementPrinciples_researchers_final.pdf

33 r4d Programme. r4d Programme [Internet]. Swiss Programme for Research on Global Issues for Development; [cited 2019 Aug 25]. Retrieved from: http://www.snf.ch/SiteCollectionDocuments/SNF_r4D_Brochure_210x175_RZ_EN_09_interactive_B_Button.pdf

34 Confédération suisse. Development and Cooperation - Focus of Research [Internet]. International Cooperation. [cited 2018 Nov 16]. Retrieved from: https://www.eda.admin.ch/deza/en/home/activities-projects/activities/research-culture/research/forschungsschwerpunkte.html

35 Factsheets of r4d programme, modules and projects 2018 [Internet]. Swiss Agency for Development and Cooperation & Swiss National Science Foundation; 2018 [cited 2019 Mar 27]. Retrieved from: http://www.r4d.ch/SiteCollectionDocuments/r4d_all_Factsheets_2018.pdf

36 r4d Programme. Additional thematic call in the r4d programme [Internet]. Swiss Programme for Research on Global Issues for Development; [cited 2019 Aug 25]. Retrieved from: http://www.r4d.ch/SiteCollectionDocuments/r4d_Call_AddThematicCall.pdf

37 Sustainable Management of Ecosystems for the Provision of Ecosystem Services [Internet]. Swiss Programme for Research on Global Issues for Development; [cited 2019 Mar 27]. Retrieved from: https://www.hes-so.ch/data/documents/r4d-call-ecosystems-2157.pdf

38 r4d Programme. Thematically Open Research [Internet]. Swiss Programme for Research on Global Issues for Development; [cited 2019 Mar 27]. Retrieved from: http://www.snf.ch/SiteCollectionDocuments/Web-News/r4d_call_141007_2nd_thematically_open_research.pdf

39 r4d Programme. Mid-term Review of the Swiss Programme for Research on Global Issues for Development [Internet]. Bern, Switzerland: Swiss Programme for Research on Global Issues for Development; 2018 Mar [cited 2019 Aug 25]. Retrieved from: http://www.r4d.ch/SiteCollectionDocuments/180328_r4d_MTR_FullReport_MgntResponse.pdf

40 Employment in the context of sustainable development [Internet]. Swiss Programme for Research on Global Issues for Development; [cited 2019 Apr 2]. Retrieved from: http://www.r4d.ch/SiteCollectionDocuments/r4d_Call_employment.pdf

41 Innovation in Agricultural and Food Systems for Food Security [Internet]. Swiss Programme for Research on Global Issues for Development; [cited 2019 Apr 2]. Retrieved from: http://www.r4d.ch/SiteCollectionDocuments/r4d_Call_food-security.pdf

42 Provision systems and financing mechanisms in the public health sector [Internet]. Swiss Programme for Research on Global Issues for Development; [cited 2019 Apr 2]. Retrieved from: http://www.snf.ch/SiteCollectionDocuments/call_pub_health_r4d.pdf

43 Causes of and Solutions to Social Conflicts in Contexts of Weak Public Institutions or State Fragility [Internet]. Swiss Programme for Research on Global Issues for Development; [cited 2019 Apr 2]. Retrieved from: http://www.r4d.ch/SiteCollectionDocuments/r4d_Call_social-conflicts.pdf

44 Third thematically open call in the r4d programme [Internet]. Swiss Programme for Research on Global Issues for Development; [cited 2019 Apr 2]. Retrieved from: http://www.r4d.ch/SiteCollectionDocuments/r4d_OC3_CallDocument_Final_allAnnexes.pdf

Global Challenges Research Fund (GCRF) and Newton Fund

45 Official Development Assistance - Global Challenges Research Fund Guidance [Internet]. Global Challenges Research Fund; [cited 2019 Mar 11]. Retrieved from: https://www.ukri.org/wp-content/uploads/2020/10/UKRI-271020-GCRFODAGuidance.pdf

46 UK Strategy for the Global Challenges Research Fund (GCRF) [Internet]. London, United Kingdom: Department for Business, Energy & Industrial Strategy; 2017 Jun [cited 2019 Mar 11]. Retrieved from: https://aka.ukri.org/files/legacy/research/gcrf-strategy-june-2017

47 Global Challenges Research Fund (GCRF): how the fund works [Internet]. Department for Business, Energy & Industrial Strategy. 2017 [cited 2019 Jan 2]. Retrieved from: https://www.gov.uk/government/publications/global-challenges-research-fund/global-challenges-research-fund-gcrf-how-the-fund-works

48 ICAI. Global Challenges Research Fund - A rapid review [Internet]. London, United Kingdom: Independent Commission for Aid Impact; 2017 Sep [cited 2019 May 28]. Retrieved from: https://icai.independent.gov.uk/wp-content/uploads/ICAI-GCRF-Review.pdf

49 BEIS & DFID. HM Government response to the Independent Commission for Aid Impact rapid review of the Global Challenges Research Fund [Internet]. Department for Business, Energy & Industrial Strategy & Department for International Development; 2017 Oct [cited 2019 May 27]. Retrieved from: https://www.gov.uk/government/publications/hm-government-response-to-the-icai-rapid-review-of-gcrf

50 Manji A, Mandler P. Parliamentary Scrutiny of Aid Spending: The Case of the Global Challenges Research Fund. Parliamentary Affairs. 2018;1–22.

51 Manji A, Mandler P. Written evidence submitted to the International Development Select Committee - UK Aid: Other Government Departments Inquiry [Internet]. 2017 Feb [cited 2019 Mar 11]. Retrieved from: http://data.parliament.uk/writtenevidence/committeeevidence.svc/evidencedocument/international-development-committee/uk-aid-other-government-departments/written/46950.pdf

52 UK aid: tackling global challenges in the national interest [Internet]. London, United Kingdom: HM Treasury & Department for International Development; 2015 Nov [cited 2019 Mar 11]. Retrieved from: https://assets.publishing.service.gov.uk/government/uploads/system/uploads/attachment_data/file/478834/ODA_strategy_final_web_0905.pdf

53 ICAI. A preliminary investigation of Official Development Assistance (ODA) spent by departments other than DFID [Internet]. Independent Commission for Aid Impact; 2015 Feb [cited 2019 May 28]. Retrieved from: https://icai.independent.gov.uk/wp-content/uploads/A-preliminary-investigation-of-Official-Development-Assistance-ODA-spe....pdf

54 BEIS. Research & Innovation: Official Development Assistance (ODA): Statement of Intent [Internet]. Department for Business, Energy & Industrial Strategy; 2017 Jun [cited 2019 May 28]. Retrieved from: https://assets.publishing.service.gov.uk/government/uploads/system/uploads/attachment_data/file/623850/beis-research-innovation-oda-statement.pdf

55 About - Newton Fund [Internet]. Newton Fund. 2015 [cited 2019 Mar 7]. Retrieved from: https://www.newtonfund.ac.uk/about/

56 Newton Fund: Frequently Asked Questions [Internet]. Newton Fund; 2019 Mar. Retrieved from:

https://assets.publishing.service.gov.uk/government/uploads/system/uploads/attachment_data/file/550747/Newton-Fund-frequently-asked-questions.pdf

57 Newton Fund - Future work plan [Internet]. Independent Commission for Aid Impact. 2019 [cited 2019 Mar 7]. Retrieved from: https://icai.independent.gov.uk/upcoming-reviews/newton-fund/

58 ICAI. A performance review approach paper [Internet]. London, United Kingdom: Independent Commission for Aid Impact; 2018 Aug [cited 2019 Mar 12]. Retrieved from: https://icai.independent.gov.uk/wp-content/uploads/ICAI-Newton-Fund-AP-1.pdf

59 The Newton Fund: Science and Innovation for Development and Diplomacy. Science & Diplomacy [Internet]. 2016 Dec [cited 2019 May 17];5(4). Retrieved from: http://www.sciencediplomacy.org/article/2016/newton-fund-science-and-innovation-for-development-and-diplomacy

60 Reddy P, Desai R, Sifunda S, Chalkidou K, Hongoro C, Macharia W, et al. “You Travel Faster Alone, but Further Together”: Learning From a Cross Country Research Collaboration From a British Council Newton Fund Grant. Int J Health Policy Manag. 2018;7(11):977–81.

61 ICAI. The Newton Fund: A performance review [Internet]. Independent Commission for Aid Impact; 2019 Jun [cited 2019 Jul 8]. Retrieved from: https://icai.independent.gov.uk/wp-content/uploads/The-Newton-Fund.pdf

62 ICAI. The current state of UK aid: A synthesis of ICAI findings from 2015 to 2019 [Internet]. Independent Commission for Aid Impact; 2019 Jun [cited 2019 Jul 8]. Retrieved from: https://icai.independent.gov.uk/report/the-current-state-of-uk-aid/
